# Scanning Electron Microscopic Assessment of Stent Coating Integrity in Jailed Wire Technique for Bifurcation Treatment

**DOI:** 10.1155/2021/2629393

**Published:** 2021-05-24

**Authors:** Lijian Gao, Ce Zhang, Huanhuan Wang, Yiqun Zhang, Zhan Gao, Bo Xu, Jue Chen, Jinqing Yuan, Shubin Qiao, Jilin Chen

**Affiliations:** ^1^Coronary Heart Disease Center, Fuwai Hospital, National Center for Cardiovascular Diseases, Chinese Academy of Medical Sciences and Peking Union Medical College, Beijing 100037, China; ^2^Abbott Vascular, Beijing 100045, China

## Abstract

**Objectives:**

To assess the impact of different guidewires on stent coating integrity in jailed wire technique (JWT) for bifurcation treatment.

**Background:**

JWT is commonly adopted to protect side branch in provisional one-stent strategy for coronary bifurcation lesions. However, this technique may cause defects in stent coatings. The degree of coating damage caused by different types of jailed wires remains unknown.

**Methods:**

A fluid model with a bifurcation was established to mimic the condition in vivo. One-stent strategy was performed with three types of guidewire (nonpolymer-jacketed wire, intermediate polymer-jacketed wire, and full polymer-jacketed wire) tested for JWT. Scanning electron microscopy (SEM) was used to evaluate stent coating integrity and wire structure. The degrees of coating defects were recorded as no, slight, moderate, and severe defects.

**Results:**

A total of 27 samples were tested. Analyses of SEM images showed a significant difference in the degree of coating damage among the three types of wire after the procedure of JWT (*P* < 0.001). Nonpolymer-jacketed wire could inevitably cause a severe defect in stent coatings, while full polymer-jacketed wire caused the least coating damages. Besides, there were varying degrees of coil deformation in nonpolymer-jacketed wires, while no surface damage or jacket shearing was observed in full polymer-jacketed wires.

**Conclusions:**

Although nonpolymer-jacketed wire has long been recommended for JWT, our bench-side study suggests that full polymer-jacketed wire may be a better choice. Further clinical studies are needed to confirm our findings.

## 1. Introduction

Coronary bifurcation lesions, defined as a coronary artery narrowing occurring adjacent to, and/or involving, the origin of a significant side branch (SB) [1], are common and account for approximately 20% of all percutaneous coronary interventions [2]. Generally, a single-stent strategy (main vessel only with a provisional approach to SB stenting) or a prespecified two-stent strategy is used for bifurcation lesions treatment, with the former being the preferred strategy [3–5].

SB occlusion can be a serious complication of provisional SB stenting strategy, which is associated with increased risks of cardiac death and myocardial infarction [6]. Thus, jailed wire technique (JWT) is commonly adopted in this setting for SB protection. However, this technique may cause defects in the polymer coatings of drug-eluting stent (DES), which can promote thrombus formation and lead to adverse clinical events [7].

There are multiple types of guide wire for SB protection, which can be broadly classified into 3 categories: nonpolymer-jacketed wire, intermediate polymer-jacketed wire, and full polymer-jacketed wire. Previous population-based studies indicated that polymer-jacketed wires were more resistant to retrieval damage and more efficient in crossing the SB ostium in JWT, compared with the nonpolymer-jacketed wires [8, 9]. Nevertheless, the degree of damage in stent coating caused by different types of jailed wires, which is closely related to clinical events, cannot be examined in vivo and remains unknown so far. In this regard, the current study utilized a scanning electron microscope (SEM) to evaluate the integrity of stent coatings with 3 different types of jailed wires in a bifurcation fluid model.

## 2. Materials and Methods

### 2.1. Model Setup and Stent Implantation

**Figure 1:**
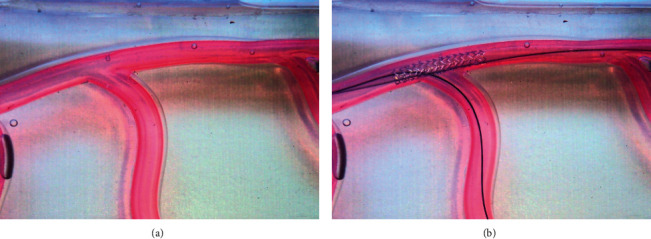
Illustrations of the fluid model without the stent and the wires (a) and with the stent and the wires (b).

A fluid model with a main vessel (inner diameter: 3.0 mm) and a SB (inner diameter: 2.0 mm) was established (Figure 1(a)). Soft materials (silicon), 37°C normal saline, and peristaltic pump were used to mimic the coronary bifurcation in humans. All models were purchased from Abbott US factory, which were made from a metal mould. The size, hardness, and quality were all the same. A LX-OO Shore OO durometer (Graigar, China) was used to measure the hardness of the silicon vessel wall. The average hardness of the wall is 70 HOO (Table 1). The inside wall of the model was totally smooth, and there was no calcification. A Xience V DES (3 × 23 mm, Abbott Vascular, Santa Clara, US) was implanted in the main vessel of each model (Figure 1(b)).

JWT was adopted in the SB. Briefly, two guide wires were inserted to the distal main branch and SB, respectively. The stent was then deployed in the main vessel at nominal pressure (10 atm). Finally, the wire in the SB, which was “jailed” by the stent, was pulled from underneath the stent struts.

### 2.2. Jailed Wire

Three types of guide wire were used as jailed wires: (1) Runthrough NS guidewire (Terumo, Tokyo, Japan) with coiled tip and multiple kinds of coating but no polymer jacket, (2) Hi-Torque Balance Middleweight Universal II Guidewire (HT BMW Universal II, Abbott Vascular, Santa Clara, US) with coiled head and polymer-jacketed body (intermediate polymer-jacketed), and (3) Hi-Torque Whisper Guidewire (HT Whisper, Abbott Vascular, Santa Clara, US) with full polymer jacket. Each type of wire was tested by 9 samples.

### 2.3. Scanning Electron Microscopy

Once the procedure was performed, the silicon vessel was dissolved with boiled water. The stent was then taken out and scanned immediately using a SEM (S4800, Hitachi, Tokyo, Japan), a technique which allows close examination of the coating surface of stent, after removal of the jailed wire, by an independent investigator who was blinded to the grouping. The stent coating integrity and wire structure of each sample were evaluated using highly magnified SEM images. The definitions of coating defect were as follows: (1) no defect, full polymer integrity with no struts exposed; (2) slight defects, struts exposed <100 *μ*m; (3) moderate defects: 100 *μ*m ≤ struts exposed < 200 *μ*m; (4) severe defects: struts exposed ≥200 *μ*m.

### 2.4. Statistical Analysis

Summary statistics were presented as frequency and percentage for category variables. Fisher's exact test was performed for comparison. Two-sided *P* values of <0.05 were considered statistically significant. Analyses were performed using SPSS software version 22.0 (IBM, Armonk, NY, USA).

## 3. Results

### 3.1. Defect in Stent Coatings

**Figure 2:**
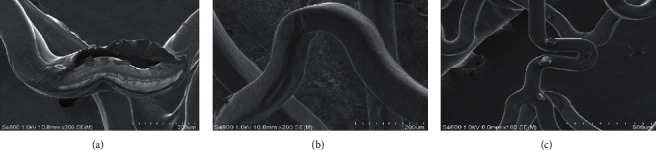
Representative SEM images of stent coating integrity after the procedure of JWT from the Runthrough NS wire group (a), HT BMW Universal II wire group (b), and HT Whisper wire group (c).

A total of 135 SEM images of DES were carefully examined, demonstrating different impacts of the three types of wire on coating integrity of DES (Figure 2). Runthrough NS wires caused the greatest damages to stent coatings among the three types of wire, with severe defects occurring in all test samples (9/9, 100%, Table 2). Slight (4/9, 44%) to moderate (4/9, 44%) coating defects were observed in the HT BMW Universal II wire group, which were significantly less severe than those in the Runthrough NS wire group. The degree of coating damages was lowest in the HT Whisper wire group, with no moderate or severe defects.

### 3.2. Defect in Guidewire

**Figure 3:**
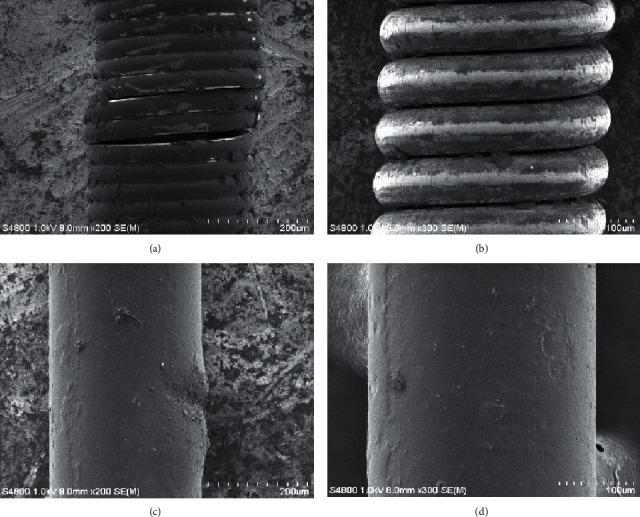
Representative SEM images of coil structure of Runthrough NS wire group (a), coil structure of the HT BMW Universal II wire group (b), jacket structure of the HT BMW Universal II wire group (c), and jacket structure of the HT Whisper group (d).

A total of 16 SEM images of guidewire were carefully examined. Structure integrity was distinctly different among the three types of guidewire (Figure 3). There were varying degrees of coil deformation in the Runthrough NS wire group, while the coil and jacket remained structurally intact in the HT BMW Universal II wire group. Neither surface damage nor jacket shearing was observed in the HT Whisper wire group.

## 4. Discussion

In this bench-side study, we found that nonpolymer-jacketed wire could inevitably cause severe defect in stent coatings in the procedure of JWT, while full polymer-jacketed wire caused the least coating damages. Besides, there was no surface damage or jacket shearing observed in full polymer-jacketed wire.

Coronary bifurcation lesion has been challenging in interventional cardiology because it is important to preserve physiologic blood flow in SB while optimizing stent implantation in the main vessel. It has a lower procedural success rate and increased rates of long-term adverse cardiac events compared with nonbifurcation lesion [10]. Meta-analyses suggested that provisional SB stenting strategy was associated with reduced long-term all-cause mortality compared with two-stent strategy for bifurcation lesion treatment [3–5]. Therefore, provisional SB stenting strategy should be considered the preferred approach for most cases, as also recommended by the European Bifurcation Club [11].

SB protection is necessary for provisional SB stenting strategy, including JWT and jailed balloon technique. A recent study reported that jailed microcatheter technique was also feasible but with higher cost due to additional devices such as the 7F guiding catheter and microcatheter [12]. Among these techniques, JWT with coiled wire is more often used in clinical practice. Despite its advantage of maximum lubricity which makes it easy to be withdrawn from a jailed position, polymer-jacketed wire is usually not recommended for JWT due to the concern over jacket shearing and even fracture during extraction [13]. In our study, we did not observe any jacket shearing of full polymer-jacketed wire, suggesting its safety for JWT.

Considerable attention has been tied to the impact of the coating defect on thrombogenicity. On the one hand, it can cause local drug missing and abnormal flow, which enhances thrombogenicity [14]. On the other hand, drug overdosage can occur elsewhere, leading to delayed endothelialization of the stent struts, which is associated with an increased risk of stent thrombosis probably due to prolonged contact between blood and DES [15]. For the first time, our study compared the degree of coating defects caused by 3 common types of guidewire in the procedure of JWT and found that full polymer-jacketed wire caused the least coating damages with no significant shearing of the polymer jacket. This finding raises a question about the optimal choice of guidewire in JWT, since coiled wire has been recommended for a long time [16].

There are some limitations that should be noted. First, the study was based on a simple, in vitro model with no lesions, e.g., plaque or calcification, which did not exactly mimic the conditions in vivo. Second, the jailed wires were all pulled back by manpower rather than a motorized pullback equipment. However, all experiments were performed by the same technician. Therefore, the force of pulling the wire back was roughly the same across each experiment, which could reduce operational differences among the experiments to some extent. Third, three kinds of products commonly used in clinical practice were tested in this study. The results might not be generalized to other products of each type. Fourth, the findings from this bench-side study should be interpreted cautiously, and further clinical trials were needed to confirm them.

## 5. Conclusion

Among the three types of guidewire tested in our study, nonpolymer-jacketed coiled wire inevitably causes severe defects to the stent coating in the procedure of JWT, while full polymer-jacketed wire causes the least coating damage without any jacket shearing. Coiled wire has long been recommended for SB protection. However, based our findings, full polymer-jacketed wire may be a better choice.

## Figures and Tables

**Table 1:** Hardness measurement of the silicon vessel wall.

Testing time	Testing results, HOO
1	69
2	70
3	70
4	71
5	70

**Table 2:** Impact of the three types of wire on coating integrity in the procedure of JWT.

	Total (*n* = 27)	Runthrough NS wire (*n* = 9)	HT BMW universal II wire (*n* = 9)^#^	HT whisper wire (*n* = 9)^#^	*P* value^*∗*^
No defect	3 (11.1)	0 (0.0)	1 (11.1)	2 (22.2)	<0.001
Slight defects	11 (40.7)	0 (0.0)	4 (44.4)	7 (77.8)	
Moderate defects	4 (14.8)	0 (0.0)	4 (44.4)	0 (0.0)	
Severe defects	9 (33.3)	9 (100.0)	0 (0.0)	0 (0.0)	

^*#*^ *P* < 0.001 compared with the Runthrough NS wire group.  ^*∗*^Comparison among three groups.

## Data Availability

The clinical data used to support the findings of this study are available from the corresponding author upon request.
